# Robust background modelling in *DIALS*

**DOI:** 10.1107/S1600576716013595

**Published:** 2016-10-21

**Authors:** James M. Parkhurst, Graeme Winter, David G. Waterman, Luis Fuentes-Montero, Richard J. Gildea, Garib N. Murshudov, Gwyndaf Evans

**Affiliations:** a Diamond Light Source Ltd, Harwell Science and Innovation Campus, Didcot OX11 0DE, UK; b Laboratory of Molecular Biology, Francis Crick Avenue, Cambridge CB2 0QH, UK; c STFC Rutherford Appleton Laboratory, Didcot OX11 0FA, UK; d CCP4, Research Complex at Harwell, Rutherford Appleton Laboratory, Didcot OX11 0FA, UK

**Keywords:** integration, robust outlier rejection, generalized linear models, background modelling

## Abstract

A method for estimating the background under each reflection during integration that is robust in the presence of pixel outliers is presented. The method uses a generalized linear model approach that is more appropriate for use with Poisson distributed data than traditional approaches to pixel outlier handling in integration programs. The algorithm is most applicable to data with a very low background level where assumptions of a normal distribution are no longer valid as an approximation to the Poisson distribution. It is shown that traditional methods can result in the systematic underestimation of background values. This then results in the reflection intensities being overestimated and gives rise to a change in the overall distribution of reflection intensities in a dataset such that too few weak reflections appear to be recorded. Statistical tests performed during data reduction may mistakenly attribute this to merohedral twinning in the crystal. Application of the robust generalized linear model algorithm is shown to correct for this bias.

## Abstract 2

The application of a robust generalized linear model framework for the modelling of reflection backgrounds in X-ray diffraction images is described.

## 1. Introduction   

In macromolecular crystallography (MX), integration programs – such as *MOSFLM* (Leslie, 1999 ▸), *XDS* (Kabsch, 2010 ▸), d*TREK (Pflugrath, 1999 ▸) and *DIALS* (Waterman *et al.*, 2013 ▸) – are used to estimate the intensities of individual Bragg reflections from a set of X-ray diffraction images. Whilst details of the processing differ, these programs all follow the same basic procedure to calculate the intensity estimates. For each reflection, pixels in the neighbourhood of the predicted Bragg peak are labelled as either ‘foreground’ or ‘background’ pixels through the application of a model of the shape of the reflection on the detector. The reflection intensity may be estimated by subtracting the sum of the estimated background values from the sum of the total number of counts in the foreground region. This is termed ‘summation integration’. The background in the foreground region is unknown and is therefore estimated from the surrounding background pixels assuming smooth variation in the background counts.

An accurate estimate of the background is a prerequisite for deriving an accurate estimate of the reflection intensity. Integration programs typically assume that the background in the vicinity of a reflection peak can be modelled either as a constant value (Kabsch, 2010 ▸) or as a plane with a small gradient (Leslie, 1999 ▸). Since the reflection peak typically extends across an area containing a small number of pixels, these assumptions generally hold true and the resulting simple models have the advantage of being computationally inexpensive to calculate from the surrounding background pixels.

The situation is complicated by the presence of pixels whose values appear not to be drawn from the same distribution as other pixels in the background region assuming the simple background model. Typically these pixels contain a higher number of counts relative to their neighbours than would be expected if they were drawn from the same distribution. The counts in these pixels can be the result of, for example, hot pixels (defective pixels which always show a large number of counts), zingers (random unmodelled spikes in intensity from, for example, cosmic rays), intensity from adjacent reflections, ice rings or other unmodelled intensity. Background estimation routines in integration programs need to be resistant to such outlier pixels. Therefore these programs implement methods to exclude outliers from the background calculation.

In this paper we compare the use of different outlier handling methods within the *DIALS* framework and introduce a method based on generalized linear models. The *DIALS* framework allows the user to choose from one of several simple algorithms as well as implementations of methods used in other integration packages. The following methods have been implemented in *DIALS*:

(1) *null*. No outlier handling is used.

(2) *truncated*. This method excludes extreme pixel values by discarding a fraction of the pixels (by default 5%) containing the highest and lowest number of counts.

(3) *nsigma*. This method excludes extreme pixel values by computing the mean and standard deviation (σ) of the pixel values and computing a threshold such that all pixels with values outside 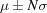 are discarded, where the default value for parameter *N* is 3. In our implementation, the procedure is applied once; however, an alternative approach may be to apply the procedure iteratively.

(4) *tukey*. Extreme pixel values are excluded by computing the median and interquartile range (IQR). Pixels with values 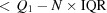 and values 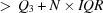 are discarded, where the default value for *N* is 1.5.

(5) *plane*. This is an implementation of the method used in *MOSFLM* (Leslie, 1999 ▸). The authors were fortunate to have access to the *MOSFLM* source code and were therefore able to verify that the algorithm implemented in *DIALS* gave equivalent results. First a percentage of the highest-valued pixels are discarded and a plane is computed from the remaining background pixels such that the modelled background at each pixel position (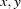) is 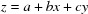, where the origin of *x* and *y* is at the peak position. The value of *a* is, therefore, the mean background. Then all pixels are checked and discarded if their absolute deviation from the plane 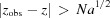, where the default value for *N* is 4.

(6) *normal*. This is an implementation of the method described by Kabsch (2010 ▸). The method assumes that the pixel values in the background region are approximated by a normal distribution. The pixels are sorted by increasing value and their distribution checked for normality. The highest-valued pixels are then iteratively removed until the distribution of the remaining pixels is approximately normal. It should be noted that the authors did not have access to the *XDS* source code that implements this method so were unable to verify that the algorithm implemented in *DIALS* gave equivalent results. Additionally, newer versions of *XDS* adapted for low-background data use a different method (Diederichs, 2015 ▸).

(7) *glm*. The robust generalized linear model (GLM) algorithm described in this paper.

Most of the methods for handling outliers described above rely on the assumption that the pixel values are drawn from a normal distribution, whereas in reality the data are Poisson distributed. As the mean expected value increases, a Poisson distribution is well approximated by a normal distribution; however, as the mean tends towards zero, this approximation becomes increasingly inappropriate. Therefore, although successfully used for data collected on CCD detectors, traditional methods may have problems when used on data collected on photon counting detectors such as the Dectris Pilatus (Henrich *et al.*, 2009 ▸). Data collected using these detectors are associated with having a lower background than data collected on CCD detectors. This is partly due to the opportunity for collecting increasingly fine φ-sliced data offered by these detectors because of the fast readout and reduced noise associated with them (Mueller *et al.*, 2012 ▸). Additionally, new beamlines have been designed where the whole experiment, including the sample and detector, is within a vacuum (Wagner *et al.*, 2016 ▸). Data from these beamlines are associated with very low background owing to the absence of scattering by the air. The design of beamlines has also contributed to the ability to collect data with lower background. Evans *et al.* (2011 ▸) showed how, for small crystals, matching the beam size to the size of the crystal could result in a drastic reduction in the X-ray background by reducing the volume of non-diffracting material that the X-rays impinge upon.

Intuitively, outlier handling methods which remove values purely from one side of the distribution will result in a biased estimate of the Poisson mean. Since the Poisson distribution is asymmetric, simple outlier handling techniques which remove a fixed percentage of pixels from either side (as in the truncated method described above) may also introduce bias. The bias for the truncated estimator of the Poisson mean is given below: 
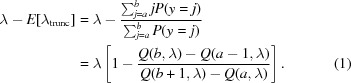



Here 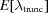 is the expected value of the truncated estimator and 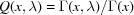 is the regularized gamma function. The bias of the estimator is dependent on the Poisson mean λ. In the case of the non-truncated estimate of the mean of a Poisson distribution, 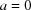 and 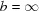. 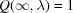 and 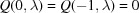; therefore the bias of the non-truncated estimator is zero. Note that any method which attempts to remove outliers from the data will systematically reduce the variance of the distribution even when no outliers are present.

In this paper, it is shown how the use of inappropriate outlier handling methods can lead to poor background determination and systematic bias in the estimated background level. The use of a simple robust estimation method using generalized linear models where the pixel values are explicitly assumed to be drawn from a Poisson distribution is proposed. It is shown that use of this algorithm results in superior statistical behaviour compared to existing algorithms.

## 2. Algorithm   

### 2.1. Generalized linear models   

Generalized linear models, first described by Nelder & Wedderburn (1972 ▸), are a generalization of ordinary linear regression. In linear regression, the errors in the dependent variables are assumed to be normally distributed. Generalized linear models extend this to allow the errors in the dependent variables to be drawn from a range of distributions in the over-dispersed exponential family, including the Poisson distribution. In the generalized linear model framework, the linear predictor, 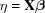, is related to the distribution *via* a link function, 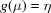. Here, 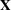 is the design matrix – a matrix of the explanatory variables – and 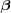 is a vector of the model parameters. In the case of the Poisson model, the link function is the natural logarithm, so that 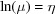. The maximum likelihood estimate is typically found using iteratively reweighted least squares. This is done as it is computationally flexible and allows a numerical solution to be found when it is difficult to maximize the likelihood function directly.

### 2.2. Robust estimation   

A method to apply robust estimation to the generalized linear model framework is described by Cantoni & Ronchetti (2001 ▸). The maximum likelihood function is replaced by a quasi-likelihood estimator whose score function, 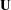, is given by 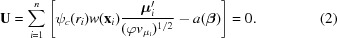



Here, 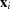 is a row of the design matrix, 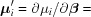 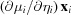 and 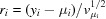 are the Pearson residuals for each observation, 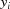, with respect to its expected value 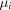 and variance 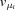. φ is the dispersion, which, for a Poisson distribution is known to be equal to 1. The functions 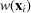 and 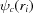 provide weights on the explanatory variables and dependent variables, respectively. Here, since it is assumed that each pixel has identical weighting, the weights for the explanatory variables, *x*, are set to 1 [*i.e.* 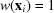]. The weighting on the dependent variables, 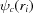, gives the estimator its robust characteristics. In this application of the algorithm, the Huber weighting function is used, as described by Cantoni & Ronchetti (2001 ▸) and shown below: 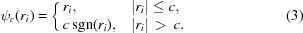



This weighting function has the effect of damping values outside a range defined by the tuning constant, *c*. A value of 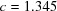 is used, corresponding to an efficiency of 95% for a normal distribution (Heritier *et al.*, 2009 ▸). The efficiency of an estimator is a measure of how optimal the estimator is relative to the best possible estimator. Increasing the value of the tuning parameter increases the efficiency of the estimator but decreases its robustness to outliers. A value of 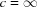 results in the same estimator as for the normal GLM approach.

The constant 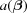 is a correction term used to ensure Fisher consistency; *i.e.* the correction term ensures that an estimate based on the entire population, rather than a finite sample, would result in the true parameter value being obtained (Fisher, 1922 ▸). The estimator is said to be Fisher consistent if 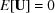. The correction term is computed simply by expanding 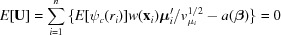 and is given by
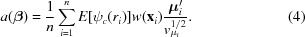



The algorithm was implemented in C++ for use within *DIALS*. It is available in the *GLMTBX* package within the *cctbx* library (Grosse-Kunstleve *et al.*, 2002 ▸). In this implementation, the parameter estimates are obtained by solving equation (2) using iteratively reweighted least squares as described by Cantoni & Ronchetti (2001 ▸) and Heritier *et al.* (2009 ▸). The equations for asymptotic variance of the estimator given by Cantoni & Ronchetti (2001 ▸, Appendix *B*) and Heritier *et al.* (2009 ▸, Appendix *E*.2) contain an error (Cantoni, 2015 ▸). For completeness, a description of the algorithm, including corrections, is provided in Appendix *A* [App appa].

### 2.3. Background models   

In applying the GLM approach to modelling of the background, instead of modelling the expected background as a constant or a plane, the logarithm of the expected background is modelled as a constant or a plane. Note that, for a constant background model, the two are equivalent. The rows of the design matrix for the constant and planar models are 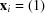 and 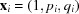, respectively, where 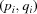 is the coordinate of the *i*th pixel on the detector.

Since the algorithm will be applied to each reflection in the dataset independently and a typical X-ray diffraction dataset contains many reflections (a high-multiplicity dataset may have >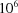 reflections), there is a requirement for the algorithm to be computationally efficient. Since the background models being used are very simple, the general algorithm can be simplified. Appendix *B* [App appb] provides a simplification of the general algorithm in the case of the constant background model.

## 3. Analysis   

### 3.1. Experimental data   

In order to evaluate the effect that different outlier handling methods have on the quality of the processed data, three datasets were selected.

(1) A weak thaumatin dataset collected on Diamond beamline I04 and available online (Winter & Hall, 2014 ▸). This dataset was chosen as it is a standard test dataset used by the *DIALS* development team. The average background over all resolution ranges is less than 1 count per pixel. In addition, it has a low incidence of outliers in the background pixels; an outlier handling algorithm should also be able to handle a good dataset without degrading it. The dataset was processed to a resolution of 1.2 Å.

(2) A ruthenium polypyridyl complex bound to duplex DNA (Hall *et al.*, 2011 ▸) collected at Diamond beamline I02 and available online (Winter & Hall, 2016 ▸). This dataset was chosen because of the presence of a number of outliers in the background that were observed to cause the wrong point group to be found in the downstream data processing. The dataset was processed to a resolution of 1.2 Å. The average background is around 2.5 counts per pixel at low resolution but decreases rapidly at high resolution.

(3) A weak thermolysin dataset collected on Diamond beamline I03 and available online (Winter & McAuley, 2016 ▸). This dataset was chosen because it is extremely weak, with an average intensity of less than 0.15 counts per pixel across the whole resolution range. Additionally, it was observed that some data processing programs gave poor results for these data, which was attributed to the poor handling of the low background. The dataset was processed to a resolution of 1.5 Å.

**Figure 1:**
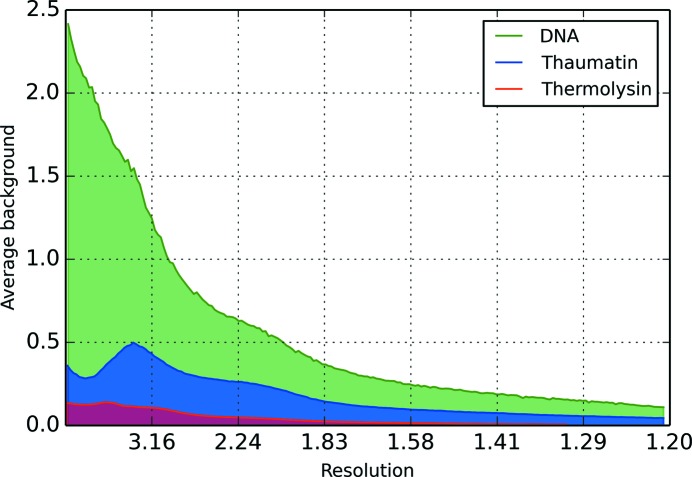
The average background level across the resolution range for each dataset.

**Figure 2:**
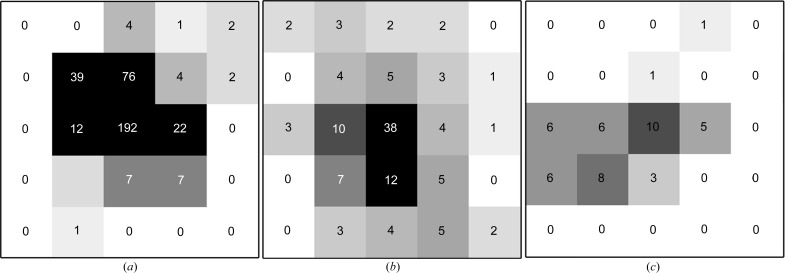
An example reflection shoebox with pixel values, observed at 3 Å, for (*a*) thaumatin, (*b*) DNA and (*c*) thermolysin.

The average background pixel value, binned by resolution, for each dataset can be seen in Fig. 1 ▸. Additionally, a randomly selected spot, observed at 3 Å, is shown for each dataset in Fig. 2 ▸; in each case, the background is primarily composed of pixels with 0 or 1 counts in them. Any algorithm which assumes a normal distribution of pixel values is likely to perform badly on these data.

### 3.2. Data analysis   

Each dataset was processed with *xia2* (Winter, 2010 ▸) using *DIALS* (Waterman *et al.*, 2013 ▸) as the data analysis engine. Subsequent data reduction was performed in *xia2* using the programs *POINTLESS* (Evans, 2006 ▸), *AIMLESS* (Evans & Murshudov, 2013 ▸) and *CTRUNCATE* (Winn *et al.*, 2011 ▸). Identical data processing protocols were used for each dataset with the exception of the choice of outlier handling method. Details of how data processing was performed are given in Appendix *C* [App appc].

### 3.3. Background estimates   

**Figure 3:**
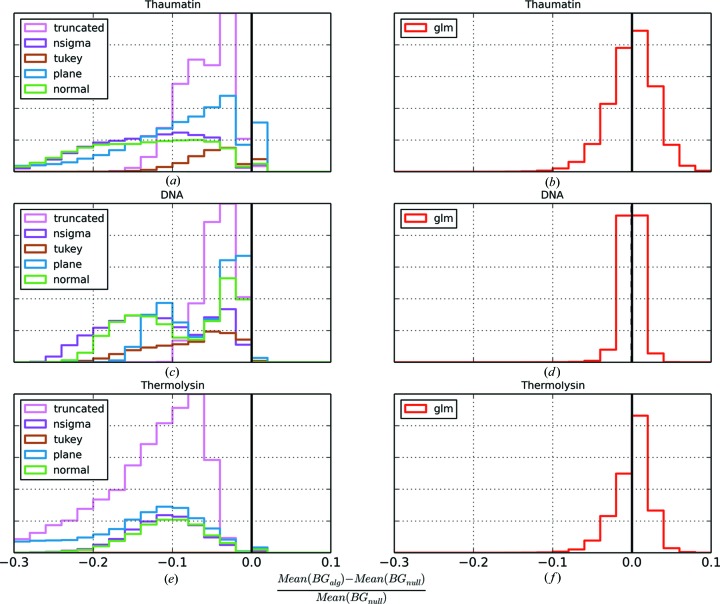
Histograms of normalized differences between the mean background with outlier handling for each outlier algorithm and the mean background with no outlier handling. For clarity, the plots for the GLM method are shown separately. The vertical black line indicates zero difference between the estimates. The estimates using the GLM algorithm are distributed more symmetrically around the null estimates, while all the other algorithms show significant systematic bias in the estimated background levels.

In general, for well measured data, pixel outliers in the background region should only affect a minority of reflections. This is the case for the three datasets considered here, where most reflections are free from pixel outliers in the background region. It is expected, therefore, that for the majority of reflections the background estimates using a well behaved outlier handling algorithm should be comparable to those using no outlier handling. Fig. 3 ▸ shows histograms of the normalized difference in background estimates with and without outlier handling for five existing methods and the GLM approach adopted here.

It can be seen that the traditional outlier handling methods introduce negative bias into the background estimate; the background level is systematically lower than that using no outlier handling. Of additional concern is a feature shown in Table 1 ▸. This gives the percentage of reflections whose background is estimated as exactly zero owing to all nonzero valued pixels in the background being rejected by the outlier handling algorithm. For some of the algorithms, particularly when applied to the very weak thermolysin dataset, this percentage is very high, indicating that for low background levels the algorithm is rejecting all nonzero pixels as outliers. In contrast, for the GLM method, it can be readily seen that the background estimates show significantly less systematic bias in the background level than seen for the other methods. On average the background estimates resulting from the GLM methods are approximately equal to those with no outlier handling. The mean normalized difference between the estimates from the GLM method and the estimates with no outlier handling are 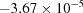, 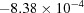 and 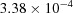 for the thaumatin, DNA and thermolysin datasets, respectively.

To test the behaviour of the GLM method in the presence of outlier pixels, we selected Bragg reflections whose background regions contained outliers as follows. Reflections whose background pixels have an index of dispersion 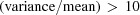 were selected and on this basis 15 out of 389 442 reflections were chosen for the thaumatin dataset, 60 of out 219 431 for the DNA dataset and 272 out of 3 322 808 for the thermolysin dataset. For Poisson distributed data, the index of dispersion should be equal to 1 [with a variance of 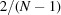, where *N* is the sample size]; values much greater than 1 indicate that the pixel values are over-dispersed relative to a Poisson distribution. This indicates that the pixel values are not all drawn from the same distribution and thus provides a reasonable, straightforward, method of selecting reflections with potential pixel outliers.

**Figure 4:**
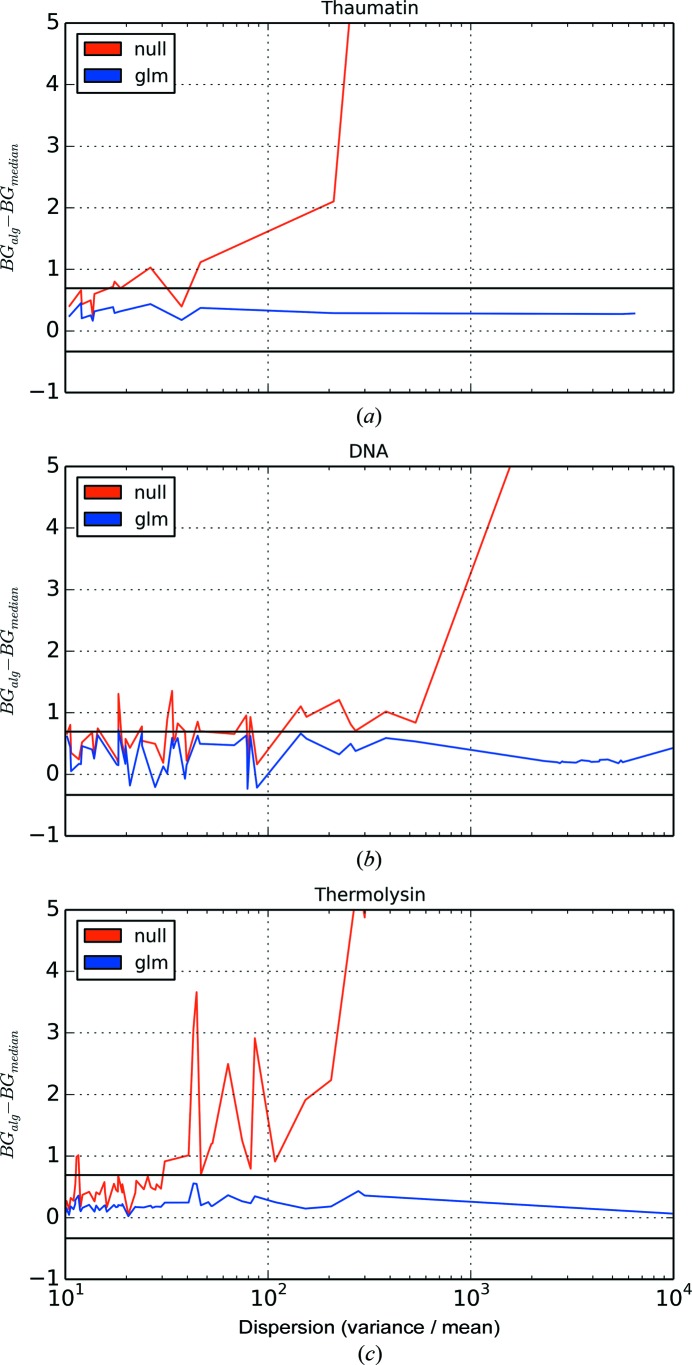
The difference between the estimated background value either with no outlier handling or with the GLM algorithm, and the median (*i.e.* most robust) background estimate for Bragg reflections with large indices of dispersion in the surrounding background pixels (an indication of the presence of pixel outliers) for (*a*) thaumatin, (*b*) DNA and (*c*) thermolysin. The horizontal black lines in each plot are at 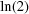 and 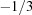; for a Poisson distribution, the bounds on the median are 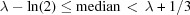 (Choi, 1994 ▸).

Fig. 4 ▸ shows the difference between the estimated background and the median background value (*i.e.* the most robust estimate of the background) for no outlier handling and for the GLM method. Note that whilst the median is the most robust estimate, in the sense that it is the estimate of central tendency least susceptible to outliers, it is not appropriate for use here since, for very low background, the median is likely to be equal to zero and the background will be systematically underestimated. However, for a Poisson distribution with rate parameter λ, the bounds of the median are 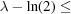 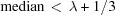 (Choi, 1994 ▸); a robust estimate of the background level should be within these bounds. As expected, with no outlier handling, the background estimate is vastly overestimated for increasing index of dispersion. With the robust GLM algorithm, the estimated background value is within the bounds given by the median background value, indicating that the algorithm is adequately handling outliers.

### 3.4. Effects on data reduction   

Since the background values are systematically underestimated for many of the algorithms, the intensities of the reflections are systematically overestimated. This impacts on the distribution of observed reflection intensities, resulting in the appearance of too few weak reflections being recorded. This can cause problems with statistics that test for twinning in the data (Yeates, 1997 ▸). Two such statistics are the L test (Padilla & Yeates, 2003 ▸) and the moments test (Stein, 2007 ▸). Table 2 ▸ shows the twin fractions resulting from application of the two twinning tests as implemented in *CTRUNCATE* for each dataset and for each outlier handling algorithm. Table 2 ▸ shows that, in most cases, the traditional outlier handling algorithms introduce, to varying degrees, the appearance of twinning. In contrast, for the data processed with no outlier handling, and for the GLM method, this effect is consistently absent.

**Figure 5:**
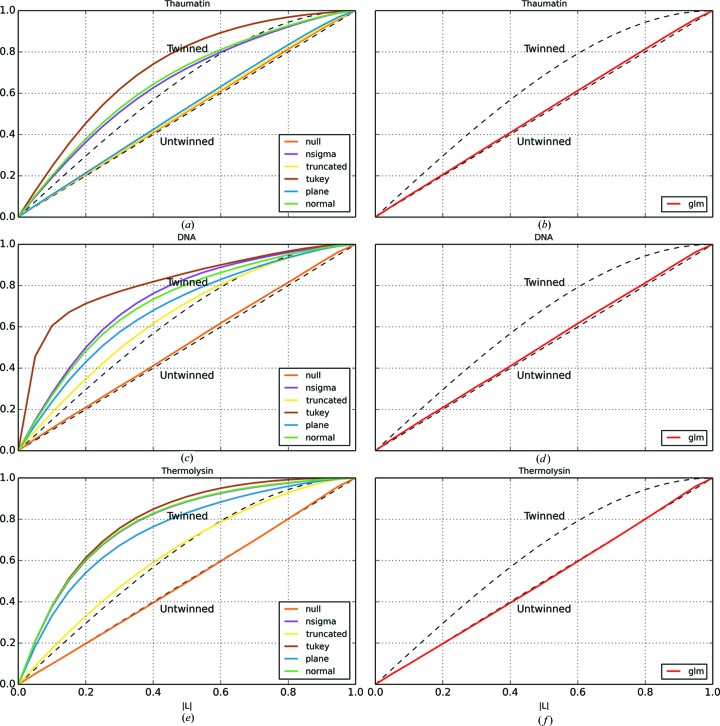
Cumulative distribution function for 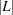 for thaumatin with (*a*) the traditional outlier handling methods and (*b*) the GLM method, for DNA with (*c*) the traditional outlier handling methods and (*d*) the GLM method, and for thermolysin with (*e*) the traditional outlier handling methods and (*f*) the GLM method.

**Figure 6:**
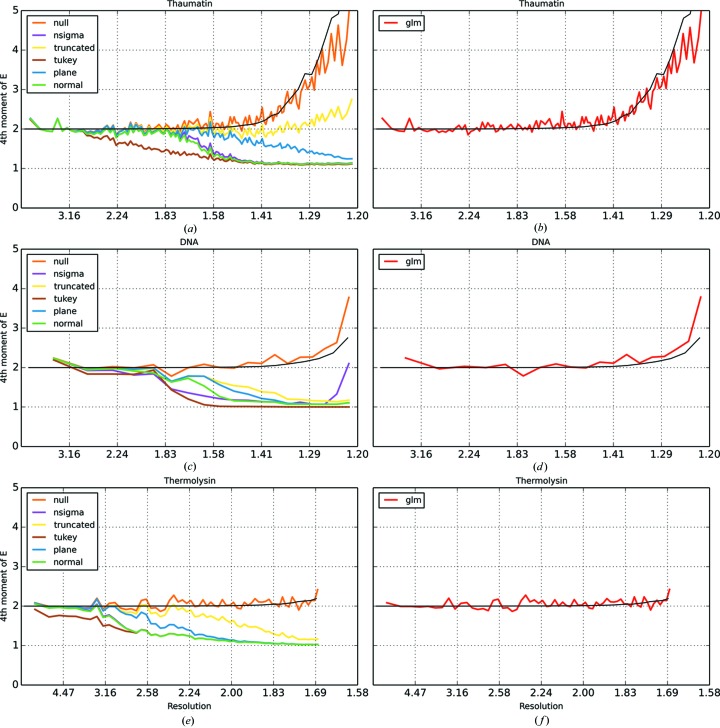
Fourth acentric moment of *E versus* resolution for thaumatin with (*a*) the traditional outlier handling methods and (*b*) the GLM method, for DNA with (*c*) the traditional outlier handling methods and (*d*) the GLM method, and for thermolysin with (*e*) the traditional outlier handling methods and (*f*) the GLM method. The theoretical curve for the acentric moments is shown in black.

The impact on the distribution of intensities is illustrated in more detail by Figs. 5 ▸ and 6 ▸. Fig. 5 ▸ shows the cumulative distribution function for 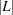 as produced by *CTRUNCATE* for each dataset and each outlier handling method. For clarity, the results from the GLM algorithm are shown in a separate plot in each case. Fig. 6 ▸ shows the fourth acentric moments of *E*, the normalized structure factors, against resolution for each dataset processed with each outlier handling method.

For error-free data, the fourth acentric moment of the normalized structure factors at low resolution tends towards a value of 2 for untwinned data and 1.5 for perfectly twinned data (Stein, 2007 ▸). When the variances on the intensities are taken into account, the value of the moment is inflated by 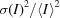. This is shown by the black theoretical curve in Fig. 6 ▸; this curve was generated by the *PHASER* program (McCoy *et al.*, 2007 ▸). Here we can see that, as the resolution increases, the data based on traditional methods show a reduced spread in the distribution of intensities, which may be interpreted as increasing amounts of twinning. In reality, the plot probably results from a dual effect. The background level decreases at high resolution, so the effect of the bias in the background estimates becomes increasingly pronounced. At the same time, the intensity of the reflections also decreases at high resolution, meaning that the relative effect of the systematically lower background estimates is amplified. In contrast, the GLM method shows the expected behaviour. At low resolution, the fourth moment is around 2, indicating no twinning. At high resolution, the moments increase as expected owing to the decreasing signal-to-noise ratio; the increase follows the theoretical curve.

## 4. Conclusion   

The use of a robust generalized linear model algorithm for the estimation of the background under the reflection peaks in X-ray diffraction data has been presented. Traditional methods for handling pixel outliers systematically underestimate the background level and consequently overestimate the reflection intensities even in the absence of any pixel outliers in the raw data. This can cause statistical tests to give the false impression that a crystal is twinned. The GLM method used here is robust against such effects. When no outliers are present, the estimates given by the GLM algorithm are, on average, the same as those with no outlier handling; the mean normalized difference between the estimates derived from the GLM method and those with no outlier handling are 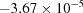, 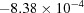 and 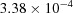 for the thaumatin, DNA and thermolysin datasets, respectively. When outliers are present, the method gives values within the expected bounds of the median. The method is implemented in *DIALS* and is currently the default algorithm when run standalone or through *xia2*.

## Figures and Tables

**Table 1:** The percentage of reflections (%) where all nonzero pixels were rejected by the outlier handling algorithm resulting in a background estimate of zero counts per pixel

	Thaumatin	DNA	Thermolysin
*truncated*	0.0	0.0	0.0
*nsigma*	31.3	0.9	76.3
*tukey*	77.9	56.8	95.0
*plane*	0.7	0.0	30.2
*normal*	37.0	0.0	78.2
*glm*	0.0	0.0	0.0

**Table 2:** The twin fractions deduced from the L and fourth moments tests reported by *CTRUNCATE* for each dataset processed using each outlier handling algorithm

	Thaumatin		DNA		Thermolysin	
Algorithm	L test	4th moments	L test	4th moments	L test	4th moments
*truncated*	0.04	0.00	0.50	0.28	0.50	0.23
*nsigma*	0.50	0.27	0.50	0.50	0.50	0.50
*tukey*	0.50	0.50	0.50	0.50	0.50	0.50
*plane*	0.06	0.01	0.50	0.42	0.50	0.50
*normal*	0.50	0.30	0.50	0.50	0.50	0.50
*glm*	0.03	0.00	0.04	0.00	0.03	0.00
*null*	0.03	0.00	0.05	0.00	0.03	0.00

**Table 3:** Definition of mathematical quantities used

Item	Definition
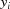	The value of the *i*th pixel.
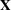	The design matrix describing the generalized linear model. A row in the design matrix is given as 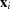 ; each row gives the explanatory variables for pixel *i*.
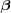	The vector of model parameters which are estimated from the quasi-likelihood algorithm.
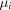	The estimated Poisson mean for the *i*th pixel, computed from the model as 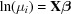 .
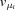	The variance for the *i*th pixel. For a Poisson distribution this is equal to the mean, 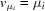 .
φ	The dispersion. For a Poisson distribution, 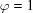 .
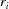	The residual for the *i*th pixel given by 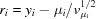 .
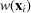	The weights on each row of the design matrix. In our implementation these weights are equal to 1.
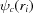	The weights on the residuals as defined in equation (3).
*c*	The tuning constant specifying the robustness of the algorithm. Smaller values increase the robustness of the algorithm.
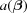	The Fisher consistency correction as defined in equation (4).
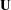	The scoring function for the quasi-likelihood estimator.
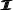	The Fisher information matrix.

**Table 4:** The parameters required to invoke a particular background algorithm in *DIALS*

Algorithm	Parameters
*truncated*	integration.background.algorithm=simple
	integration.background.simple.outlier.algorithm=truncated
*nsigma*	integration.background.algorithm=simple
	integration.background.simple.outlier.algorithm=nsigma
*tukey*	integration.background.algorithm=simple
	integration.background.simple.outlier.algorithm=tukey
*plane*	integration.background.algorithm=simple
	integration.background.simple.outlier.algorithm=plane
*normal*	integration.background.algorithm=simple
	integration.background.simple.outlier.algorithm=normal
*null*	integration.background.algorithm=simple
	integration.background.simple.outlier.algorithm=null
*glm*	integration.background.algorithm=glm

## Appendix A Robust GLM algorithm implementation in *DIALS*

For convenience, the terms used in the following equations are defined again in Table 3 ▸.

The background, , at each pixel is estimated from the generalized linear model as . Given initial model parameter estimates , the parameter estimate for the next iteration of the algorithm, , is given by

The scoring function, , is given by

The only additional term that needs to be calculated here is the expectation . In order to compute this, let us denote  and . For a Poisson distribution

The expectation, , is then given by

The Fisher information matrix, , is given by

The diagonal components of the matrix  are given by

For a Poisson distribution,   and  . The expectation is given by

## Appendix B Simplified algorithm for constant background model

In the case of the constant background model (*i.e.* where a robust estimate of the mean of the background pixels is calculated), the model only has a single parameter, β, and the rows of the design matrix, , are all defined as . The estimate of the background is then  and the iterative algorithm to estimate the model parameter, β, is simplified to the following:

Since the expectations  and   do not depend on , and  is the same for each point, they are constant for a given value of μ as shown below:

The scoring function, *U*, and the Fisher information, , are then simplified to the following:

The updated value of the parameter estimate  is then given by

## Appendix C Program operation

The command line parameters needed to invoke each method are listed in Table 4 ▸. To set these parameters through *xia2*, they should be saved to a file (*e.g.* parameters.phil) and *xia2* called as follows:

# Call XIA2 with DIALS

xia2 -dials\

dials.integrate.phil_file=parameters.phil\

image=image_0001.cbf
